# Reply from Dr. Shailendra Verma

**Published:** 2006-04

**Authors:** Shail Verma

**Affiliations:** Melanoma Disease Site Group, Cancer Care Ontario Practice Guidelines Initiative, Program in Evidence-based Care, McMaster University, 3rd Floor, 1280 Main Street West, Hamilton, Ontario L8S 4L8

Thank you for your comments. We acknowledge and concur with your opinion regarding the assertion that high-dose interferon is associated with a significant survival benefit. Our guideline has been corrected to reflect this, and the latest iteration states that “Our review of the available literature identified no systemic adjuvant therapy that confers a significant survival benefit in patients with high-risk resected primary melanoma. However high-dose interferon treatment should be considered in such patients as such therapy is associated with significant improvement in disease-free survival and reduction in 2-year mortality.”

